# Systemic Sclerosis Serum Significantly Impairs the Multi-Step Lymphangiogenic Process: In Vitro Evidence

**DOI:** 10.3390/ijms20246189

**Published:** 2019-12-07

**Authors:** Mirko Manetti, Eloisa Romano, Irene Rosa, Bianca Saveria Fioretto, Serena Guiducci, Silvia Bellando-Randone, Erika Pigatto, Franco Cozzi, Lidia Ibba-Manneschi, Marco Matucci-Cerinic

**Affiliations:** 1 Section of Anatomy and Histology, Department of Experimental and Clinical Medicine, University of Florence, 50134 Florence, Italy; irene.rosa@unifi.it (I.R.); lidia.ibba@unifi.it (L.I.-M.); 2 Section of Rheumatology, Department of Experimental and Clinical Medicine, University of Florence, 50134 Florence, Italy; eloisaromano@libero.it (E.R.); biancafioretto@icloud.com (B.S.F.); serena.guiducci@unifi.it (S.G.); silvia.bellandorandone@unifi.it (S.B.-R.); marco.matuccicerinic@unifi.it (M.M.-C.); 3 Division of Rheumatology and Scleroderma Unit, Department of Geriatric Medicine, Azienda Ospedaliero-Universitaria Careggi (AOUC), 50134 Florence, Italy; 4 Rheumatology Unit, Department of Medicine—DIMED, University of Padova, 35128 Padova, Italy; erika.pigatto@gmail.com (E.P.); franco.cozzi@unipd.it (F.C.); 5 Internal Medicine Unit, Ospedale Classificato Villa Salus, 30174 Venice, Italy

**Keywords:** systemic sclerosis, scleroderma, dermal lymphatic microvascular endothelial cells, lymphangiogenesis, VEGFR-3/Flt-4, NRP-2

## Abstract

In systemic sclerosis (SSc), the possible involvement of lymphatic microcirculation and lymphangiogenesis has traditionally been overshadowed by the greater emphasis placed on dysfunctional blood vascular system and angiogenesis. In the present in vitro study, we explore for the first time whether the SSc microenvironment may interfere with lymphangiogenesis, a complex, multi-step process in which lymphatic microvascular endothelial cells (LMVECs) sprout, migrate, and proliferate to generate new lymphatic capillaries. Normal human adult dermal LMVECs from three donors were treated with serum from SSc patients (*n* = 8), serum from healthy individuals (*n* = 8), or recombinant human vascular endothelial growth factor (VEGF)-C as a positive control for lymphangiogenesis. Cell proliferation, Boyden chamber Matrigel chemoinvasion, wound healing capacity, and lymphatic capillary morphogenesis on Geltrex were assayed. VEGF-C serum levels were measured by enzyme-linked immunosorbent assay. Gene and protein expression levels of the lymphangiogenic orchestrators VEGF receptor-3 (VEGFR-3)/Flt-4 and neuropilin-2 (NRP-2) were determined by real-time PCR and Western blotting, respectively. Conditioning with SSc serum significantly inhibited LMVEC proliferation, Matrigel invasion, and wound healing capacity with respect to healthy serum. The ability of LMVECs to form lymphatic tubes on Geltrex was also severely compromised in the presence of SSc serum. VEGF-C levels were comparable in SSc and healthy sera. Treatment with SSc serum resulted in a significant downregulation of both VEGFR-3/Flt-4 and NRP-2 mRNA and protein levels. In SSc, the pathologic environment severely hampers every lymphangiogenesis step, likely through the reduction of pro-lymphangiogenic VEGFR-3/NRP-2 co-receptor signaling. The impairment of the lymphangiogenic process opens a new scenario underlying SSc vascular pathophysiology, which is worth investigating further.

## 1. Introduction

The pathogenic scenario of systemic sclerosis (SSc, scleroderma) is dominated by peripheral microvascular damage and immune system disturbances, culminating in progressive fibrosis of the skin and internal organs [1,2]. Early endothelial cell activation/injury and failure in vascular recovery are considered the hallmarks of the disease, and may lead to a variety of clinical manifestations, ranging from Raynaud’s phenomenon and digital painless swelling (“puffy fingers”) to digital ulcers or even gangrene of the extremities in the most severe cases [3,4,5].

In SSc, great emphasis has traditionally been placed on blood vessel dysfunction and defective angiogenesis and vascular repair, while the involvement of the lymphatic vascular counterpart has been mostly overlooked [5,6,7,8]. Nonetheless, a few clinical and histopathologic studies have demonstrated that clinically-affected SSc skin displays lymphatic microvascular abnormalities, including a severe reduction in both superficial and deep dermal microlymphatic networks that has been correlated with the presence of fingertip ulcers and linked to cutaneous disease progression from an early edematous stage toward overt fibrotic remodeling [9,10,11,12]. Indeed, it has been suggested that dermal lymphatic microangiopathy may take place as a consequence of blood capillary leaking in very early SSc, when “puffy fingers” are a clinical hallmark [5]. Specifically, dermal blood capillary leaks result in greater amounts of fluid and macromolecules in the interstitium, followed by maximal increase in lymph flow, which provokes microlymphatic vessel overload/insufficiency and consequent tissue accumulation of protein-rich interstitial fluid, immune cells, chemokines, and growth factors, clinically manifesting as skin edema [5]. The ensuing inflammatory response and fibrotic process may then aggravate microlymphatic damage in a vicious circle, similar to that occurring in chronic venous insufficiency; this further impairs skin tissue homeostasis and eventually culminates in dystrophic changes like digital ulcers [5,10,12]. In SSc, it has also recently been proposed that a deficiency in repair mechanisms might prevent lymphatic microcirculation recovery and possibly exacerbate even more lymphatic microvascular injury [13]. In particular, an impairment of lymphvasculogenesis due to a reduction in the number of circulating bone marrow-derived lymphatic endothelial progenitor cells has been reported in SSc patients with digital ulcers [13].

Besides lymphvasculogenesis, lymphangiogenesis is another process that ensures embryonic lymphatic vessel development and growth; it is reactivated during post-natal life in various pathologic conditions, such as inflammation, wound healing, and tumorigenesis [14,15,16,17]. In lymphangiogenesis, new lymphatic capillaries are formed from preexisting vessels through the sprouting of lymphatic microvascular endothelial cells (LMVECs), a well-coordinated process in which migrating tip cells sample the pericellular environment in search of guidance cues and steer the forming branches, which then proliferate and extend to form new, mature, blind-ended microlymphatics [14]. Current evidence suggests that lymphatic vessel sprouting is primarily mediated by vascular endothelial growth factor (VEGF)-C and VEGF-D through the activation of VEGF receptor-3 (VEGFR-3)/Flt-4, a tyrosine kinase receptor expressed by LMVECs [14,15,17]. Moreover, VEGF-C and VEGF-D also bind to neuropilin-2 (NRP-2), which acts as a co-receptor for VEGFR-3 to promote lymphangiogenesis [14,17,18]. At present, an impairment of lymphangiogenesis in SSc can only be hypothesized on the basis of a few descriptive studies that have reported abnormal expression patterns of some of the aforementioned lymphangiogenic regulators in the circulation and skin of SSc patients [19,20]. Therefore, the objective of our in vitro study was to investigate, for the first time, whether functional defects in the lymphangiogenic process may be part of the complex scenario underlying SSc peripheral microvasculopathy.

## 2. Results

### 2.1. Conditioning of Human Dermal Lymphatic Microvascular Endothelial Cells with Systemic Sclerosis Serum Significantly Inhibits Multiple Steps of Lymphangiogenesis In Vitro

Previous studies have extensively demonstrated that treatment with SSc serum is capable of severely impairing the angiogenic performance of dermal blood microvascular endothelial cells, and that in SSc, dysfunctional angiogenesis may make a substantial contribution to the failure of peripheral blood microcirculation recovery following recurrent ischemia–reperfusion injuries [6,21,22,23,24,25,26]. Nevertheless, whether the SSc microenvironment affects the functionality of LMVECs in sustaining the lymphangiogenic process has never been explored. Therefore, here normal human dermal LMVECs were conditioned with sera from SSc patients or healthy individuals and tested in different assays, recapitulating in vitro the multiple steps of the lymphangiogenic process (i.e., cell proliferation, matrix invasion, migration, and lymphatic capillary-like tube formation). According to the literature [27,28,29], stimulation with recombinant human VEGF-C was carried out in parallel as a positive control for lymphangiogenesis for all assays.

**Figure 1:**
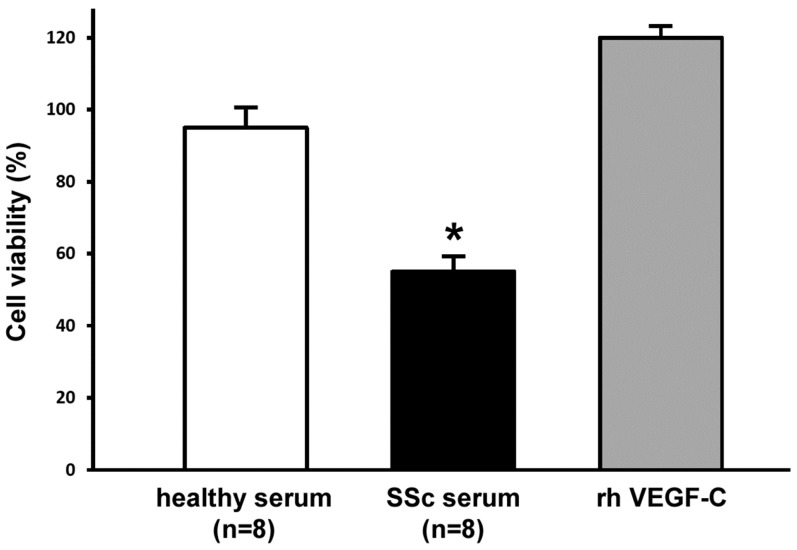
Systemic sclerosis (SSc) serum significantly inhibits proliferation of dermal lymphatic microvascular endothelial cells (LMVECs). Cell viability was measured by the WST-1 colorimetric assay after challenging the LMVECs for 48 h with serum from healthy controls (*n* = 8) or from patients with early diffuse cutaneous SSc (*n* = 8). Stimulation with pro-lymphangiogenic recombinant human (rh) vascular endothelial growth factor (VEGF)-C served as a positive control. Cell proliferation in the presence of EGM-2-MV complete medium was set as 100%; all results are normalized to this value. Data are mean ± standard error of the mean (SEM) of three independent experiments, performed in triplicate with each one of the three LMVEC lines. * *p* < 0.001 vs. healthy serum (Tukey’s test).

Conditioning with SSc serum significantly reduced LMVEC viability/proliferation compared with cells stimulated with healthy serum, as assessed by the WST-1 assay (*p* < 0.001) (Figure 1). As expected, challenging LMVECs with pro-lymphangiogenic recombinant human VEGF-C resulted in the highest proliferative response (Figure 1).

**Figure 2:**
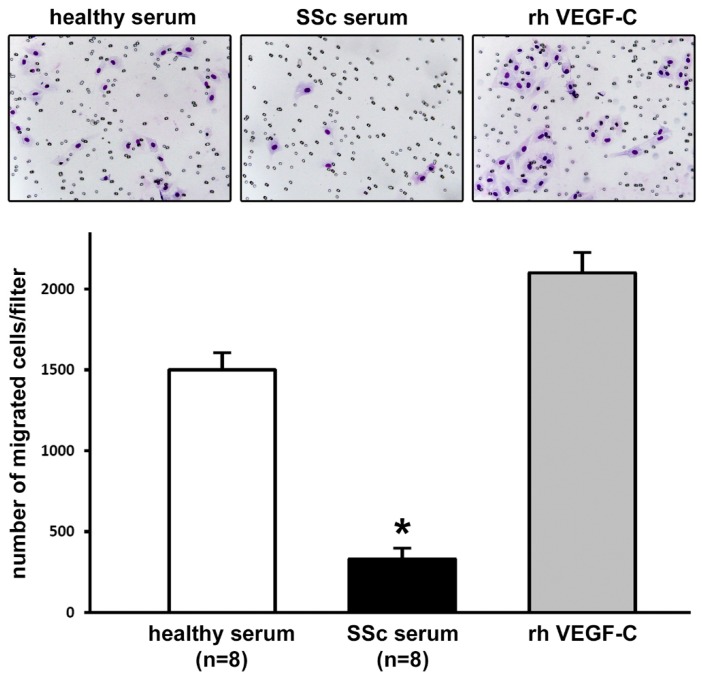
Systemic sclerosis (SSc) serum significantly impairs Matrigel chemoinvasion of dermal lymphatic microvascular endothelial cells (LMVECs). Chemoinvasion of LMVECs was tested by using the Boyden chamber assay, placing in the lower compartment healthy control sera (*n* = 8) or early diffuse cutaneous SSc sera (*n* = 8). To verify the efficiency of the assay, pro-lymphangiogenic recombinant human (rh) vascular endothelial growth factor (VEGF)-C was placed in the lower compartment in parallel experimental points (positive control). Representative images of the filters after 48 h showing invasive cells stained with Diff-Quik are shown (original magnification: ×20). The histograms show results of quantitative analysis of chemoinvasion expressed as the number of migrated cells per filter. Data are mean ± SEM of three independent experiments performed in duplicate with each one of the three LMVEC lines. * *p* < 0.001 vs. healthy serum (Tukey’s test).

We next carried out the Boyden chamber chemoinvasion assay, in order to evaluate the capability of LMVECs to invade Matrigel, which mimics the composition of the basement membrane matrix, and migrate in the surrounding space. As shown in Figure 2, the invasiveness of LMVECs was significantly inhibited in the presence of SSc serum with respect to healthy serum (*p* < 0.001). The elevated number of invasive cells detected after stimulation with recombinant human VEGF-C testified the efficiency of the assay (Figure 2).

**Figure 3:**
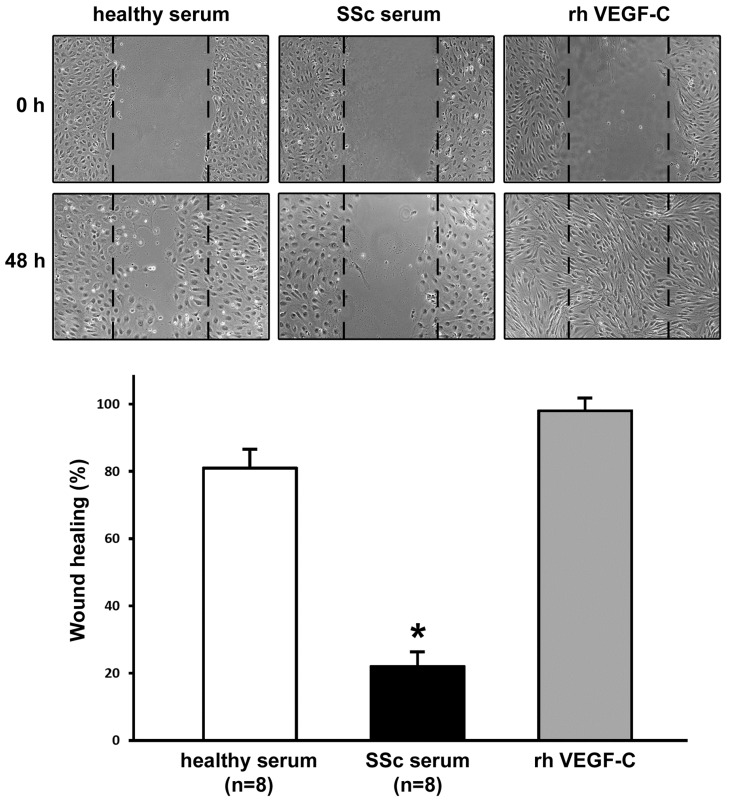
Systemic sclerosis (SSc) serum significantly impairs the wound healing capacity of dermal lymphatic microvascular endothelial cells (LMVECs). The wound healing capacity of LMVECs was determined in the presence of sera from healthy controls (*n* = 8) or patients with early diffuse cutaneous SSc (*n* = 8). To verify the efficiency of the assay, pro-lymphangiogenic recombinant human (rh) vascular endothelial growth factor (VEGF)-C was administered to LMVECs as a positive control. Representative images of the wounded area at 0 h and 48 h after scratching are shown (original magnification: ×10). The histograms show results of quantitative analysis of the percentage of wound repair. Data are mean ± SEM of three independent experiments performed in triplicate with each one of the three LMVEC lines. * *p* < 0.001 vs. healthy serum (Tukey’s test).

Cell proliferation and migration in the same experimental conditions were further assessed by using the in vitro wound healing assay. After scratching in the presence of healthy serum, LMVECs migrated into the wounded area and then proliferated, resulting into ~80% wound closure at 48 h (Figure 3). Conversely, at 48 h after scratching in the presence of SSc serum, LMVECs were unable to restore the monolayer integrity (~20% wound closure) (*p* < 0.001 vs. healthy serum-treated LMVECs) (Figure 3). An almost completely restored monolayer was observed after 48 h from wounding in cells treated with recombinant human VEGF-C (Figure 3).

**Figure 4:**
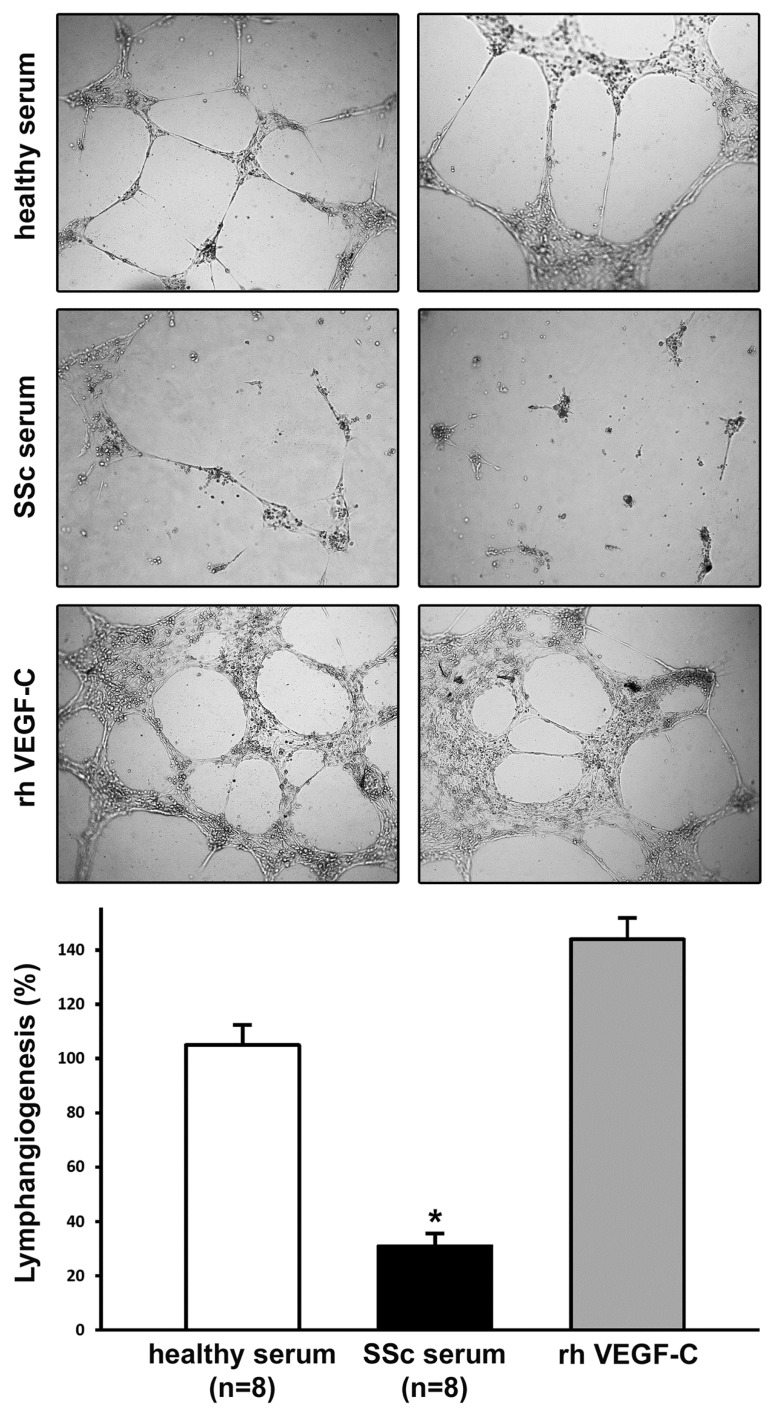
The ability of dermal lymphatic microvascular endothelial cells (LMVECs) to form capillary-like tubes on Geltrex matrix is significantly inhibited by systemic sclerosis (SSc) serum. In vitro lymphatic capillary morphogenesis was evaluated after challenge with sera from healthy controls (*n* = 8) or from patients with early diffuse cutaneous SSc (*n* = 8). Stimulation with recombinant human (rh) vascular endothelial growth factor (VEGF)-C was used as a positive control of lymphangiogenesis. Two representative images of the capillary-like tube network, formed after 48 h from LMVEC plating on Geltrex, are shown for each experimental point (original magnification: ×10). In vitro lymphangiogenesis, quantified as percent field occupancy of capillary-like tube projections, is represented in the histograms. Capillary morphogenesis of LMVECs at basal condition was set to 100%; the other results are normalized to this value. Data are mean ± SEM of three independent experiments, performed in duplicate with each one of the three LMVEC lines. * *p* < 0.001 vs. healthy serum (Tukey’s test).

In order to analyze the entire lymphangiogenic process in vitro, we finally performed the lymphatic capillary-like tube formation assay on Geltrex matrix. As displayed in Figure 4, the ability of LMVECs to form lymphatic capillaries on Geltrex was severely compromised in the presence of SSc serum (*p* < 0.001 vs. healthy serum). An extensive network of interconnecting lymphatic capillary-like tubes was evident after cell stimulation with pro-lymphangiogenic recombinant human VEGF-C (Figure 4).

### 2.2. Conditioning of Human Dermal Lymphatic Microvascular Endothelial Cells with Systemic Sclerosis Serum Significantly Downregulates Gene and Protein Expression of Pro-Lymphangiogenic VEGFR-3/Flt-4 and Its Co-receptor NRP-2

**Figure 5:**
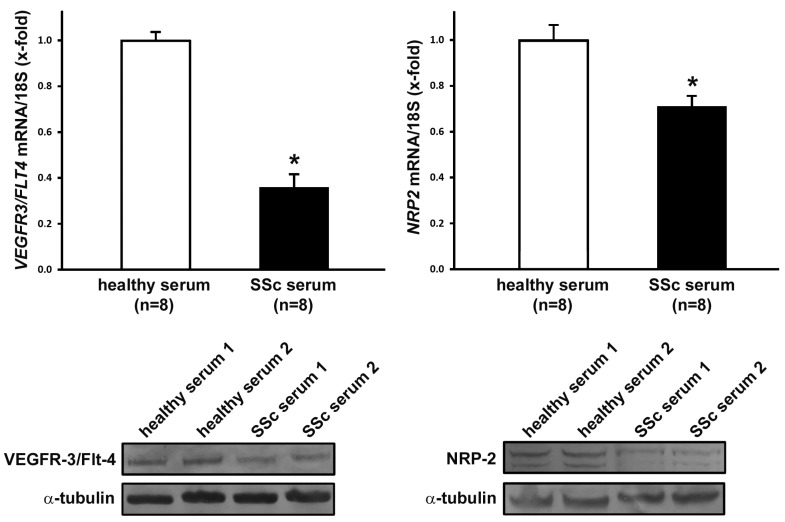
Gene and protein expression of vascular endothelial growth factor receptor-3 (VEGFR-3)/Flt-4 and neuropilin-2 (NRP-2) in dermal lymphatic microvascular endothelial cells (LMVECs) is significantly decreased by conditioning with systemic sclerosis (SSc) serum. LMVECs were treated for 48 h with sera from healthy controls (*n* = 8) or from patients with early diffuse cutaneous SSc (*n* = 8). Quantification of *VEGFR3/FLT4* and *NRP2* gene expression by real-time reverse transcription PCR is reported in the histograms. Gene expression levels in LMVECs treated with healthy serum were set to 1; the other results are normalized to this value. The reference gene used was 18S ribosomal RNA. Bars represent the mean ± SEM values of triplicate determinations from each one of the three LMVEC lines. * *p* < 0.001 vs. healthy serum (unpaired Student’s *t*-test). Representative immunoblots of VEGFR-3/Flt-4 and NRP-2 proteins are shown at the bottom; α-tubulin was measured as a loading control.

The levels of VEGF-C measured by a quantitative, colorimetric, sandwich enzyme-linked immunosorbent assay in SSc serum samples (mean ± SD values = 1.25 ± 0.21 ng/mL) were similar to those detected in healthy control serum samples (mean ± SD values = 1.08 ± 0.14 ng/mL). To shed some light on the possible mechanisms underlying the anti-lymphangiogenic effect exerted by SSc serum on LMVECs, we investigated whether it could induce any relevant changes in the expression of cell surface receptors, which act as important mediators of the pro-lymphangiogenic activity of VEGF-C. Therefore, LMVECs treated with SSc or healthy sera were analyzed for gene and protein expression levels of the pro-lymphangiogenic receptor VEGFR-3/Flt-4 and its co-receptor NRP-2 [14,30]. Conditioning of LMVECs with SSc serum resulted in a significant reduction of *VEGFR3/FLT4* and *NRP2* mRNA levels, determined by quantitative real-time PCR (*p* < 0.001 vs. healthy serum-treated LMVECs for both) (Figure 5). The downregulation of both VEGFR-3/Flt-4 and NRP-2 in LMVECs following treatment with SSc serum was confirmed at the protein level by Western blotting (Figure 5).

## 3. Discussion

The findings of this study contribute to expanding our currently limited knowledge of lymphatic vessel involvement in SSc, highlighting that impaired lymphangiogenesis may represent a previously neglected pathophysiologic factor and a potential novel therapeutic target. Indeed, we demonstrate that normal human dermal LMVECs fail to properly perform lymphangiogenesis in the presence of serum from SSc patients, which suggests that a deficiency in endogenous repair mechanisms may actively contribute to the progressive loss of the cutaneous lymphatic network previously documented in a few SSc clinical and histopathologic studies [9,10,11,12,20]. Thus, it is herein revealed for the first time that SSc-related microlymphatic dysfunction may share important pathophysiologic features with vasculopathy affecting blood microvessels, in which a prominent role for defective angiogenesis has long been established [3,6,22,31].

Lymphangiogenesis is well recognized as a highly dynamic process during embryogenesis that is subsequently largely absent under normal physiologic postnatal conditions. However, in the adult, lymphangiogenesis can take place during a variety of pathologic conditions, including inflammation, tissue repair, and tumor growth, where it mainly consists in the sprouting of new lymphatic capillaries from the preexisting ones [14,32,33]. Lymphatic capillaries are thin-walled and blind-ended endothelial tubes whose function is essential for maintaining body fluid and tissue homeostasis. Specifically, lymphatic capillaries are structurally specialized to drain blood-derived, protein-rich fluid with the clearance of macromolecules from the tissue interstitium and the formation of lymph fluid, which is transported via a network of increasingly larger vessels to the venous system [33,34]. In case of lymphatic vessel insufficiency, interstitial accumulation of macromolecules and fluid results in edema and inflammatory reactions; this rapidly evolves into fibrotic tissue remodeling, as observed in the early stage of SSc skin disease, particularly in patients with diffuse cutaneous SSc (dcSSc) [5,35]. Hence, in the present study we analyzed the lymphangiogenic performance of dermal LMVECs exposed to sera from early dcSSc patients or healthy controls to recapitulate, in vitro, a relevant pathologic or physiologic tissue microenvironment. In particular, the multiple in vitro assays employed in this work allowed an in-depth investigation of the different LMVEC functions necessary to carry out the multi-step lymphangiogenic process, such as cell survival/proliferation, invasiveness through the extracellular matrix and formation of tube-like structures. When cell viability/proliferation was specifically assessed using the WST-1 assay, our findings revealed that sera from SSc patients can exert a significant anti-proliferative effect on LMVECs, compared with healthy sera. Moreover, by using the Boyden chamber chemoinvasion assay, we exploited the capacity of LMVECs to invade the extracellular matrix and migrate in response to SSc or healthy serum, while with the wound healing assay we further evaluated cell migration and proliferation simultaneously. Of note, both LMVEC chemoinvasiveness and wound healing capacity were also significantly reduced in cultures challenged with SSc sera. Finally, the findings of capillary-like tube formation assay on Geltrex clearly revealed that the ability of LMVECs to organize three-dimensionally in an extracellular matrix was greatly compromised in the presence of SSc serum. Overall, the SSc microenvironment seems therefore capable of heavily interfering with every step of the lymphangiogenic process, in a manner rather similar to that repeatedly described for blood vessel growth/angiogenesis [23,24,25,26].

On the basis of the afore-described functional data, we next explored whether conditioning of LMVECs with SSc serum could somehow modulate the expression of the key lymphangiogenic regulator VEGFR-3/Flt-4, a receptor for both VEGF-C and VEGF-D that transduces growth, survival, and migratory signals in lymphatic endothelial cells [14,15,27,36]. Actually, we found that both mRNA and protein levels of VEGFR-3/Flt-4 were strongly downregulated in LMVECs challenged with SSc serum, compared with healthy serum-treated cells, which suggests that an impaired VEGFR-3/Flt-4-mediated signaling could be largely responsible for the observed dysfunctional lymphangiogenesis. Interestingly, a similar decrease in the expression of VEGFR-3/Flt-4 that likely contributes to a defective lymphangiogenic/lymphvasculogenic function has previously been reported in circulating lymphatic endothelial progenitor cells from SSc patients with severe peripheral vascular disease [13]. Increased *VEGFR3/FLT4* transcript levels have instead been revealed in SSc skin biopsies [20], though it should be considered that in pathologic conditions the expression of this receptor is not restricted to the lymphatic endothelium, but is found also in blood vascular endothelial cells and cells of the monocyte/macrophage lineage [37,38,39,40]. It is also interesting to note that an abnormal lymphangiogenesis with excess VEGFR-3/Flt-4-mediated signaling was previously hypothesized in SSc, because of high circulating levels of VEGF-C and VEGF-D [19,20]. However, when we measured VEGF-C levels in our serum samples, we could not find any significant difference between SSc patients and healthy controls. Thus, our current results rather suggest that, in SSc, a marked downregulation of VEGFR-3/Flt-4 may make LMVECs poorly responsive to VEGF-C-mediated, pro-lymphangiogenic stimuli. Such a scenario is further supported by the evidence that treatment of LMVECs with SSc serum also resulted in a significant downregulation of NRP-2, a key co-receptor that modulates VEGF-C/VEGFR-3 signaling in lymphatic vessel development and post-natal lymphangiogenesis [14,30,41]. In fact, it has been demonstrated that the interaction of NRP-2 with VEGFR-3 enhances VEGF-C-induced lymphatic endothelial cell survival and migration in vitro [41], and that a VEGFR-3/NRP-2 receptor complex mediates VEGF-C-induced lymphatic sprouting in vivo [30]. As a matter of fact, NRP-2 mutant mice have displayed a severe reduction of small-caliber lymphatic vessels and capillaries [42]. In SSc skin, it is therefore reasonable to suppose that a concomitant decline in the lymphatic endothelial expression of VEGFR-3/Flt-4 and NRP-2 may profoundly hamper the lymphangiogenic process, contributing to a relentless loss of the lymphatic capillary network. Furthermore, it should be considered that NRP-2 may also function as a co-receptor with the signal transducer PlexinA for class 3 semaphorins (SEMA3s), and that SEMA3F and SEMA3G have been shown to behave as negative regulators for dermal lymphangiogenesis in vivo [43]. Thus, it is clear that further in-depth studies will be necessary to clarify the precise molecular mechanisms underlying the impairment of lymphangiogenesis in SSc, including possible abnormalities in the levels of anti-lymphangiogenic SEMA3F and SEMA3G.

In summary, this study provides unprecedented evidence that the SSc pathologic milieu profoundly impairs the lymphangiogenic performance of dermal LMVECs. In perspective, we frankly acknowledge that our data represent the necessary groundwork for further investigation aimed at unveiling the likely multiple factors that, in SSc, may prevent a timely lymphangiogenic response to the ongoing destruction of the dermal microlymphatic network [9,10,11,12]. In this regard, it is noteworthy that several factors with proven anti-lymphangiogenic activity, such as transforming growth factor-β1, thrombospondin, and endostatin, are known to be increased and to have pathogenic implications in SSc [1,6,14,23,29,44,45,46,47,48]. In such a context, a very recent study has demonstrated that levels of CXCL10 and CXCL11, two C-X-C chemokines exerting angiostatic effects through their receptor CXCR3-B [49], are strongly increased in the circulation of SSc patients from the earliest disease stages, and may represent good biomarkers of vascular damage progression [50]. Interestingly, both CXCL10 and CXCL11 have been reported to potently inhibit the lymphangiogenic process [51,52], making them very attractive candidates as possible mediators of SSc-related, disturbed lymphangiogenesis, and are worthy of future investigation.

Since lymphatic vessel insufficiency in SSc skin has been suggested to broadly contribute to abnormal tissue perfusion, dystrophic changes and fibrosis [10,12], there is realistic hope that targeting impaired lymphangiogenesis might prove to be useful in preventing irreversible end-stage fibrotic damage.

## 4. Materials and Methods

### 4.1. Patients and Serum Samples

Serum samples were obtained from a total of eight patients with early dcSSc (disease duration: <2 years from first non-Raynaud symptom), fulfilling the American College of Rheumatology/European League Against Rheumatism 2013 classification criteria [53]. The patients were recruited from the Division of Rheumatology and Scleroderma Unit, Azienda Ospedaliero-Universitaria Careggi (AOUC), Florence, Italy. Patients were not on immunosuppressive medications, corticosteroids, or other disease-modifying drugs. Before the collection of peripheral blood samples, patients were washed out for 10 days from oral vasodilating drugs, and for 2 months from intravenous prostanoids. Eight age- and sex-matched healthy individuals served as controls. Demographic and clinical features of patients with SSc and healthy controls are shown in Table 1. Fresh venous blood samples were drawn and left to clot for 30 min before centrifugation at 1500× *g* for 15 min, and serum was collected and stored in aliquots at −80 °C until used for cell stimulation in different assays. The study was carried out according to the principles of the Declaration of Helsinki, and approved by the local institutional review board at the AOUC, Florence, Italy (approval number: AOUC 69/13; approval date: 17 June 2013). Written informed consent was obtained from all individuals enrolled in this study.

### 4.2. Culture of Human Lymphatic Microvascular Endothelial Cells

Three lines of adult human dermal LMVECs were purchased from Lonza (HMVEC-dLyAd; catalog no. CC-2810; Lonza, Basel, Switzerland) and routinely maintained in EGM-2-MV complete medium (EGM-2 MV Microvascular Endothelial Cell Growth Medium-2 BulletKit; catalog no. CC-3202) at 37 °C in a 5% CO_2_ incubator. These cells are at least 95% double positive for CD31/PECAM-1 and podoplanin (gp38) by flow cytometry. Once at confluence, cells were trypsinized, centrifuged, resuspended in EGM-2-MV complete medium, and seeded onto appropriate supports for the different assays.

### 4.3. Cell Viability Assay

LMVECs were seeded onto 96-multiwell plates (40 × 10^3^ cells/well) in EGM-2-MV complete medium, and were left to adhere overnight. Cells were then starved for 24 h in EBM-2 basal medium (catalog no. CC-3156; Lonza) containing 2% fetal bovine serum (FBS), and were subsequently stimulated for additional 48 h in 2% FBS–EBM-2 basal medium and 10% dcSSc serum (*n* = 8) or 10% healthy serum (*n* = 8). Stimulation with recombinant human VEGF-C (10 ng/mL; PeproTech, Rocky Hill, NJ, USA) served as a positive control. Cell viability was established by the Cell Proliferation Reagent WST-1 (4-[3-(4-iodophenyl)-2-(4-nitrophenyl)-2H-5-tetrazolio]-1,3-benzene disulfonate) colorimetric assay (Roche Diagnostics, Mannheim, Germany), according to the manufacturer’s instructions. The proliferative effect with EGM-2-MV complete medium was set as 100% proliferation. Each measurement was performed in triplicate, and the results were expressed as the percentage of increase/decrease in cell viability over the basal response.

### 4.4. Boyden Chamber Matrigel Chemoinvasion Assay

Chemoinvasion was assessed by using the Boyden chamber assay, performed in 24-multiwell plates, with inserts containing an 8 μm pore size polyethylene terephthalate (PET) membrane filter coated with Matrigel Basement Membrane Matrix (BD BioCoat Matrigel; catalog no. 354480; BD Biosciences, San Diego, CA, USA) separating the upper and lower wells of the migration chamber. The solution to be tested (750 μL of 2% FBS–EBM-2 basal medium containing 10% dcSSc serum (*n* = 8) or 10% healthy serum (*n* = 8)) was placed in the lower well, while a suspension of 25 × 10^3^ LMVECs in 2% FBS–EBM-2 basal medium was added in the upper well. To verify the efficiency of the assay, stimulation with recombinant human VEGF-C (10 ng/mL; PeproTech) was used as positive control, whereas a chemokinetic effect was excluded, using 2% FBS–EBM-2 basal medium in both the upper and the lower well (i.e., under this condition we failed to detect any cells on the lower side of the membrane). All experimental conditions were performed in duplicate. At 48 h after cell seeding, the membrane was fixed in situ for 2 min with 3% formalin in phosphate-buffered saline (PBS), and was then permeabilized for 20 min with methanol. Non-migrated cells were mechanically removed from the upper surface of the PET membrane with a cotton-tipped swab, while migrated cells, adherent on the lower filter surface, were stained for 15 min with Diff-Quik (Dade Behring, Deerfield, IL, USA). The membranes were then washed with PBS, detached from the insert with a blade, and mounted upside-down on glass slides. Each membrane was photographed under a Nikon E600 light microscope (Nikon, Tokyo, Japan) with a ×20 objective in four randomly selected fields. Migrated cells were counted in a blind manner by two independent observers, with the aid of the NIS-Elements software version 2.3 (Nikon).

### 4.5. In Vitro Wound Healing Assay

LMVECs were seeded onto 6-multiwell tissue culture plates and cultured in EGM-2-MV complete medium until 80–90% confluence. Cells were then rinsed with PBS and starved in 2% FBS–EBM-2 basal medium overnight. The next day, the medium was removed, and the monolayer was scratched with a sterile 200-μL pipette tip, in order to create a ~1 mm wide wound. After careful washing with PBS to remove detached cells, the monolayer was conditioned with 1 mL of EBM-2 medium containing 2% FBS and 10% dcSSc serum (*n* = 8), or 10% healthy serum (*n* = 8). Positive controls were obtained using EBM-2 basal medium containing 2% FBS and recombinant human VEGF-C (10 ng/mL; PeproTech), to verify the efficiency of the assay. All experimental conditions were tested in triplicate. Wound healing capacity was assessed by capturing phase-contrast images of the wounded area at the beginning and after 48 h under a Leica inverted microscope (Leica Microsystems, Mannheim, Germany) with a ×10 objective.

### 4.6. In Vitro Lymphatic Capillary-Like Tube Formation Assay

An in vitro angiogenesis assay was performed in 96-multiwell plates coated with Geltrex LDEV-Free Reduced Growth Factor Basement Membrane Matrix (catalog no. A1413202; Thermo Fisher Scientific, Waltham, MA, USA). Geltrex (50 µL/well) was allowed to polymerize for 30 min at 37 °C prior to seeding cells at the density of 14 × 10^3^ in 100 μL of 2% FBS–EBM-2 basal medium containing 10% dcSSc serum (*n* = 8), or 10% healthy serum (*n* = 8). In order to verify the efficiency of the assay (i.e., the capability of cells to form capillary-like tubes in vitro), positive controls were obtained using 2% FBS–EBM-2 basal medium supplemented with recombinant human VEGF-C (50 ng/mL; PeproTech). All experimental conditions were tested in duplicate. Plates were photographed at 48 h, and results were quantified by measuring the percent field occupancy of capillary projections, as determined by image analysis. Six to nine photographic fields from three plates were scanned for each experimental point.

### 4.7. RNA Isolation and Quantitative Real-Time PCR

Forty-eight hours after stimulation with 10% dcSSc serum (*n* = 8) or 10% healthy serum (*n* = 8), LMVECs were harvested, and the total RNA was isolated using the RNeasy Micro Kit (Qiagen, Milan, Italy). First-strand cDNA was synthesized using the QuantiTect Reverse Transcription kit (Qiagen). For mRNA quantification, SYBR Green real-time PCR was performed using the StepOnePlus Real-Time PCR System (Applied Biosystems, Milan, Italy), with melting curve analysis. Predesigned oligonucleotide primer pairs were obtained from Qiagen (QuantiTect Primer Assay). The assay identifications were Hs_FLT4_1_SG (VEGFR-3; catalog no. QT00063637), Hs_NRP2_1_SG (neuropilin-2; catalog no. QT01011794), and Hs_RRN18S_1_SG (18S ribosomal RNA; catalog no. QT00199367). Amplification was performed according to a standard protocol recommended by the manufacturer. Non-specific signals caused by primer dimers or genomic DNA were excluded by dissociation curve analysis, non-template controls, and samples without enzyme in the reverse transcription step. The 18S ribosomal RNA was measured as an endogenous control to normalize for the amounts of loaded cDNA. Differences were calculated with the threshold cycle (Ct) and comparative Ct method for relative quantification. All measurements were performed in triplicate.

### 4.8. Western Blotting

Proteins were extracted from LMVECs after conditioning with 10% dcSSc serum (*n* = 8) or 10% healthy serum (*n* = 8) for 48 h, following previously published protocols [54,55]. Thirty micrograms of total proteins were electrophoresed on NuPAGE 4 to 12% Bis-Tris Gel (Invitrogen, Carlsbad, CA, USA) and blotted onto polyvinylidene difluoride membranes (Invitrogen). The membranes were blocked with a solution included in the WesternBreeze Chromogenic Western Blot Immunodetection Kit (catalog no. WB7105; Invitrogen) for 30 min at room temperature on a rotary shaker, and were incubated for 1 h at room temperature with the following rabbit polyclonal anti-human antibodies: anti-VEGFR-3 (1:1000 dilution; catalog no. ab27278; Abcam, Cambridge, UK), anti-NRP-2 (1:1000 dilution; catalog no. ab185710; Abcam), and anti-α-tubulin (1:1000 dilution; catalog no. #2144; Cell Signaling Technology, Danvers, MA, USA), assuming α-tubulin as control invariant protein. Immunodetection was performed using the WesternBreeze Chromogenic Western Blot Immunodetection Kit protocol (Invitrogen). Band intensities were quantified with the free-share ImageJ software, 64-bit Java 1.8.0_112 Windows version (NIH, Bethesda, MD, USA; online at http://rsbweb.nih.gov/ij), and the values were normalized to α-tubulin.

### 4.9. Determination of VEGF-C Serum Levels

The levels of VEGF-C in serum samples were measured by commercial, quantitative, colorimetric, sandwich enzyme-linked immunosorbent assay, according to the manufacturer’s recommendation (eBioscience Human VEGF-C Platinum ELISA Kit; San Diego, CA, USA). The detection limit of the assay, according to the manufacturer, was 0.057 ng/mL, and the standard curve covered a concentration range from 0.23 to 15.0 ng/mL. Concentrations were calculated using a standard curve generated with specific standards provided by the manufacturer. Each sample was tested in triplicate.

### 4.10. Statistical Analysis

Statistical analyses were performed using the SPSS software for Windows, version 25.0 (Statistical Package for Social Sciences Inc., Chicago, IL, USA). Data are expressed as the mean ± standard deviation (SD) or standard error of the mean (SEM). One-way ANOVA with post-hoc Tukey’s test or unpaired Student’s *t*-test was used for statistical analyses, as applicable. Values of *p* < 0.05 were considered statistically significant.

## Figures and Tables

**Table 1:** Demographic and clinical characteristics of patients with early diffuse cutaneous systemic sclerosis (SSc) and individuals serving as healthy controls enrolled for collection of serum samples.

Characteristics	SSc Patients (*n* = 8)	Healthy Controls (*n* = 8)
Mean age, years (range)	38.5 (22–53)	38.9 (23–55)
Gender, male/female, *n*	0/8	0/8
Mean disease duration, months (range)	14.7 (9–22)	–
**Autoantibody positivity**, *n*		
Antinuclear	8	–
Anti-topoisomerase I	6	–
**Nailfold videocapillaroscopy pattern**, *n*		
Early	3	–
Active	5	–
Late	0	–
Mean modified Rodnan skin score, (range)	15.0 (10–21)	–
Interstitial lung disease ^a^, *n*	2	–

^a^ Determined by thoracic, high-resolution computer tomography.

## Abbreviations

SSc	systemic sclerosis
LMVECs	lymphatic microvascular endothelial cells
VEGF-C	vascular endothelial growth factor-C
VEGFR-3	vascular endothelial growth factor receptor-3
NRP-2	neuropilin-2
dcSSc	diffuse cutaneous systemic sclerosis
FBS	fetal bovine serum
PBS	phosphate-buffered saline
SEMA3s	class 3 semaphorins
SD	standard deviation
SEM	standard error of the mean
